# 2-Amino-5-nitro­pyridinium hydrogen selenate

**DOI:** 10.1107/S1600536809042354

**Published:** 2009-11-07

**Authors:** Samah Akriche, Mohamed Rzaigui

**Affiliations:** a Laboratoire de Chimie des Matériaux, Faculté des Sciences de Bizerte, 7021 Zarzouna Bizerte, Tunisia

## Abstract

There are two cations and two anions in the asymmetric unit of the title compound, C_5_H_6_N_3_O_2_^+^·HSeO_4_^−^. In the crystal, there are two independent chains of HSeO_4_^−^ anions running along the *a* axis, linked by O—H⋯O hydrogen bonds. Ribbons of cations linked by N—H⋯O hydrogen bonds run along the *b*-axis direction, and are further hydrogen bonded to the anions by N—H⋯O and C—H⋯O links, generating a three-dimensional network.

## Related literature

For related structures of 2-amino-5-nitro­pyridinium salts, see: Pécaut *et al.* (1993*a*, ▶ *b* ▶); Masse & Zyss (1991 ▶); Zyss *et al.* (1993 ▶); Watanabe *et al.* (1993 ▶); Pécaut & Masse (1994 ▶). For hydrogen bonds, see: Desiraju (1991 ▶); Steiner (1993 ▶, 1994 ▶). For bond lengths in related structures, see: Aakeröy *et al.* (1998 ▶). Ferraris & Ivaldi (1984 ▶).
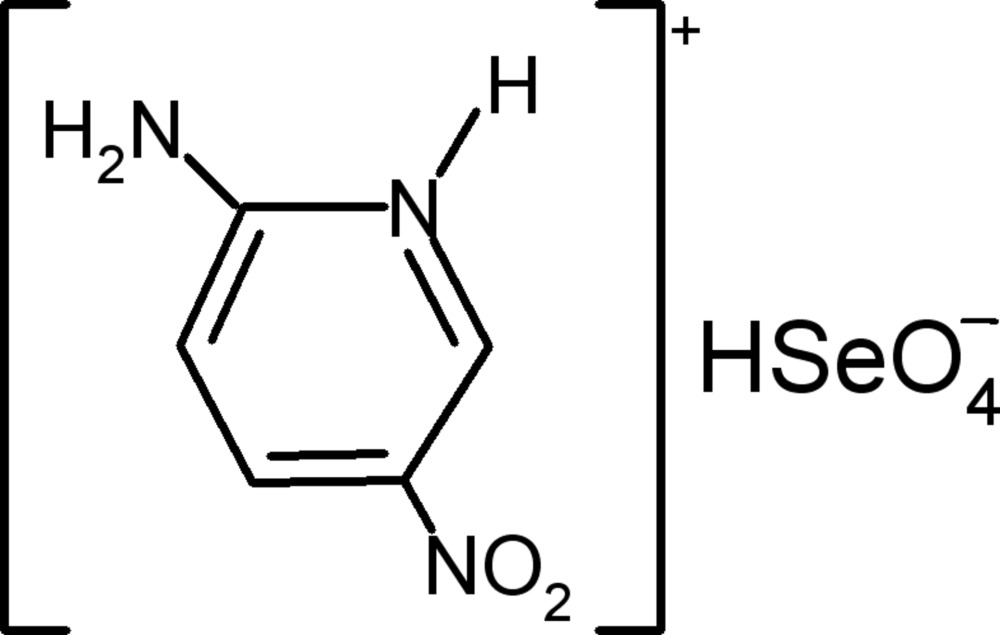


## Experimental

### 

#### Crystal data

C_5_H_6_N_3_O_2_^+^·HSeO_4_^−^
*M* *_r_* = 284.10
Orthorhombic, 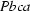

*a* = 9.092 (3) Å
*b* = 13.416 (2) Å
*c* = 30.149 (4) Å
*V* = 3677.5 (14) Å^3^
*Z* = 16
Mo *K*α radiation
μ = 4.10 mm^−1^
*T* = 298 K
0.23 × 0.21 × 0.19 mm

#### Data collection

Enraf–Nonius TurboCAD-4 diffractometer
Absorption correction: multi-scan (Blessing, 1995 ▶) *T*_min_ = 0.403, *T*_max_ = 0.444
8480 measured reflections
4426 independent reflections
2650 reflections with *I* > 2σ(*I*)
*R*_int_ = 0.075
2 standard reflections frequency: 120 min intensity decay: 6%

#### Refinement

*R*[*F* ^2^ > 2σ(*F* ^2^)] = 0.043
*wR*(*F* ^2^) = 0.101
*S* = 0.97
4426 reflections
273 parameters
H-atom parameters constrained
Δρ_max_ = 0.59 e Å^−3^
Δρ_min_ = −0.60 e Å^−3^

### 

Data collection: *CAD-4 EXPRESS* (Enraf–Nonius, 1994 ▶); cell refinement: *CAD-4 EXPRESS*; data reduction: *XCAD4* (Harms & Wocadlo, 1995 ▶); program(s) used to solve structure: *SHELXS97* (Sheldrick, 2008 ▶); program(s) used to refine structure: *SHELXL97* (Sheldrick, 2008 ▶); molecular graphics: *ORTEP-3* (Farrugia, 1997 ▶) and *DIAMOND* (Brandenburg & Putz, 2005 ▶); software used to prepare material for publication: *WinGX* (Farrugia, 1999 ▶).

## Supplementary Material

Crystal structure: contains datablocks I, global. DOI: 10.1107/S1600536809042354/hb5132sup1.cif

Structure factors: contains datablocks I. DOI: 10.1107/S1600536809042354/hb5132Isup2.hkl

Additional supplementary materials:  crystallographic information; 3D view; checkCIF report

## Figures and Tables

**Table 1:** Hydrogen-bond geometry (Å, °)

*D*—H⋯*A*	*D*—H	H⋯*A*	*D*⋯*A*	*D*—H⋯*A*
O2—H2⋯O4^i^	0.82	1.75	2.527 (5)	158
O8—H8⋯O7^ii^	0.82	1.73	2.546 (5)	173
N1—H1⋯O3	0.86	1.95	2.773 (5)	161
N2—H2*A*⋯O3	0.86	2.46	3.152 (6)	138
N2—H2*B*⋯O6	0.86	2.15	2.943 (6)	152
N2—H2*B*⋯O9^iii^	0.86	2.54	3.057 (6)	119
N4—H4⋯O5	0.86	2.01	2.769 (5)	146
N5—H5*A*⋯O5	0.86	2.27	2.958 (6)	137
N5—H5*B*⋯O1	0.86	2.02	2.833 (6)	157
N5—H5*B*⋯O11^iv^	0.86	2.56	3.016 (6)	115
C2—H2*C*⋯O6	0.93	2.37	3.132 (6)	139
C8—H8*C*⋯O4^v^	0.93	2.37	3.261 (6)	159
C3—H3⋯O7^vi^	0.93	2.41	3.202 (6)	143
C5—H5*C*⋯O2^i^	0.93	2.50	3.245 (6)	137
C5—H5*C*⋯O10^vii^	0.93	2.50	3.150 (6)	128
C7—H7⋯O1	0.93	2.52	3.228 (6)	134
C10—H10⋯O5^ii^	0.93	2.23	3.130 (6)	162

Symmetry codes: (i) 
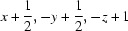
; (ii) 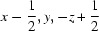; (iii) 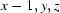; (iv) 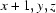; (v) 
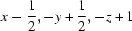
; (vi) 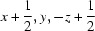; (vii) 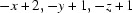.

**Fig. 1.**
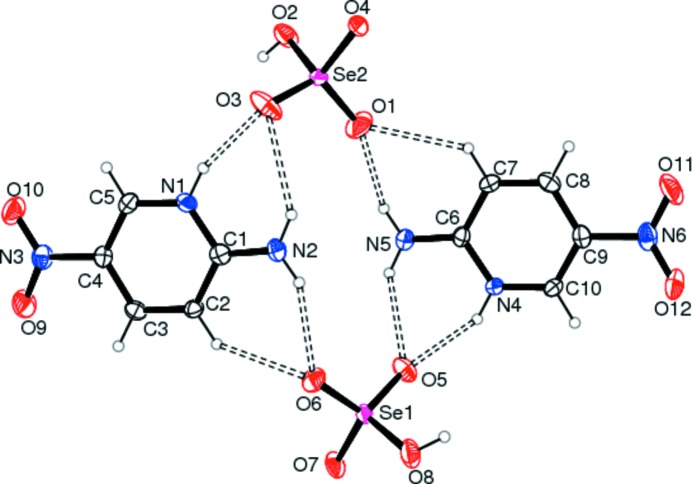
A view of (I) with displacement ellipsoids drawn at the 30% probability level. H atoms are represented as small spheres of arbitrary radii. Hydrogen bonds are represented as dashed lines.

**Fig. 2.**
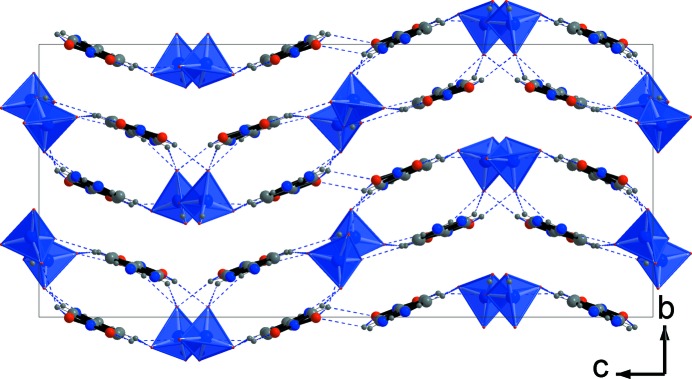
Projection of (I) along the a axis.

## supplementary crystallographic information

### Comment

The 2-amino-5-nitropyridine (2 A5NP) chromophore is promising candidate for non linear optics. From this molecule, several salts having noncentrosymmetric structures were obtained: dihydrogenphosphate, dihydrogenarsenate, chloride, bromide, tartrate, and acetophosphonate of 2-amino-5-nitropyridinium (Pécaut *et al.*, 1993a,b; Masse *et al.*, 1991; Pécaut *et al.*, 1993, Zyss *et al.*, 1993; Watanabe *et al.*, 1993; Pécaut Masse, 1994). In the framework of our systematic research on nitropyridine chromophore, we report on the new compound (C_5_H_6_N_3_O_2_)^+^, HSeO_4_^-^ synthesized from the 2-amino-5-nitropyridine and selenic acid.

The asymmetric unit of the title compound (I) that contains two 2-amino-5-nitropyridinium cations and two hydrogen selenate anions, is shown in Fig. 1. The connection between theses independent components generate a three-dimensional supramolecular network which is stabilized by hydrogen bonds, Van Der Waals and electrostatic interactions. In fact, The two hydrogen selenate anions are connected through strong hydrogen bonds characterized by relatively short distances, from 1.73 to 1.75 A% (Table 1), to form two independent robust chains extending along a direction (Fig. 2). Both cations are arranged in ribbons and anchored onto both adjacent anionic chains *via* N—H···O and C—H···O hydrogen bonds. The C—H···O bonds have already been evidenced by several authors in molecular crystals; (Desiraju *et al.*, 1991; Steiner *et al.*, 1993 and 1994). With regards to the organic subnetwork, each 2 A5NP cation is hydrogen bonded to symmetry-equivalent 2 A5NP cation by rather long N—H···O and C—H···O bonds (with distances N2—H2B···O9 (*x* - 1, *y*, *z*) = 2.54A% and C5—H5C···O10 (-*x* + 2, -*y* + 1, -*z* + 1) = 2.50 A%) as to form ribbons running along the *b* axis. In the selenate chains, it is noteworthy that the O···O distances involved in hydrogen bonds (2.527 (5) to 2.546 (5) A%) are of the same order of magnitude as the O···O distances in HSeO_4_ (2.41 to 2.56A%); this should allow us to consider the (HSeO_4_^-^)_n_ subnetwork as a polyanion. The geometrical features of HSeO_4_ entities, show that the Se—O bonds are significantly shorter [1.592 (4) to 1.623 (3) A% than the Se—OH bonds [1.690 (4) to 1.696 (4)A%], which is in accordance with the data relative to the protonated oxoanions as reported by (Ferraris *et al.*, 1984) Bond lengths and angles of the organic cations can be regarded as normal and are comparable with values of other 2-amino-5-nitropyridinium compounds. The organic ring atoms of both independent cations are essentially planar (the deviations from least-square planes are 0.001 and 0.002 Å). The angles between the plane of the NO_2_ group and the pyridinium rings are 4.9 (3) and 5.8 (4)°. This distortion is evident because the oxygen atoms of the NO_2_ group are the seat of various types of inter-and intramolecular hydrogen bonds. Moreover, the C—NH_2_ (1.312 (6) and 1.315 (6)Å) and C—NO_2_ (1.459 (6) and 1.466 (6) Å) distances in the 2 A5NP cations are respectively shortened and lengthened with respect to the C—NH_2_ (1.337 (4) Å) and C—NO_2_ (1.429 (4) Å) observed in the 2-amino-5-nitropyridine molecular crystal (Aakeröy, *et al.*, 1998). All the 2-amino-5-nitropyridinium cations hosted in various organic or inorganic matrices show the same changes in C—NH_2_ and C—NO_2_ distances, revealing a weak increase of π bond character in C—NH_2_ and a decrease in C—NO_2_.

### Experimental

The starting materials, 2-amino-5-nitropyridine (2-A5NP) and selenic acid (Aldrich, 40 wt% in H_2_O, 99.95%) were used as supplied. 5 mmol of selenic acid was added to a hot solution (20 ml of water and 5 ml of ethanol) of 2-A5NP (5 mmol). The mixture was cooled and slowly evaporated at room temperature for several days until it resulted in yellow prisms of (I).

### Crystal data

**Table:**

C_5_H_6_N_3_O_2_^+^·HO_4_Se^−^	*F*(000) = 2240
*M**_r_* = 284.10	*D*_x_ = 2.052 Mg m^−^^3^
Orthorhombic, *P**b**c**a*	Mo *K*α radiation, λ = 0.71073 Å
Hall symbol: -P 2ac 2ab	Cell parameters from 25 reflections
*a* = 9.092 (3) Å	θ = 9–11°
*b* = 13.416 (2) Å	µ = 4.10 mm^−^^1^
*c* = 30.149 (4) Å	*T* = 298 K
*V* = 3677.5 (14) Å^3^	Prism, yellow
*Z* = 16	0.23 × 0.21 × 0.19 mm

### Data collection

**Table:**

Enraf–Nonius TurboCAD-4 diffractometer	2650 reflections with *I* > 2σ(*I*)
Radiation source: Enraf Nonius FR590	*R*_int_ = 0.075
graphite	θ_max_ = 28.0°, θ_min_ = 2.6°
Non–profiled ω scans	*h* = −10→11
Absorption correction: multi-scan (Blessing, 1995)	*k* = 0→17
*T*_min_ = 0.403, *T*_max_ = 0.444	*l* = 0→39
8480 measured reflections	2 standard reflections every 120 min
4426 independent reflections	intensity decay: 6%

### Refinement

**Table:**

Refinement on *F*^2^	Primary atom site location: structure-invariant direct methods
Least-squares matrix: full	Secondary atom site location: difference Fourier map
*R*[*F*^2^ > 2σ(*F*^2^)] = 0.043	Hydrogen site location: inferred from neighbouring sites
*wR*(*F*^2^) = 0.101	H-atom parameters constrained
*S* = 0.97	*w* = 1/[σ^2^(*F*_o_^2^) + (0.0438*P*)^2^] where *P* = (*F*_o_^2^ + 2*F*_c_^2^)/3
4426 reflections	(Δ/σ)_max_ < 0.001
273 parameters	Δρ_max_ = 0.59 e Å^−^^3^
0 restraints	Δρ_min_ = −0.59 e Å^−^^3^

### Special details

**Table:**

Geometry. All e.s.d.'s (except the e.s.d. in the dihedral angle between two l.s. planes) are estimated using the full covariance matrix. The cell e.s.d.'s are taken into account individually in the estimation of e.s.d.'s in distances, angles and torsion angles; correlations between e.s.d.'s in cell parameters are only used when they are defined by crystal symmetry. An approximate (isotropic) treatment of cell e.s.d.'s is used for estimating e.s.d.'s involving l.s. planes.
Refinement. Refinement of *F*^2^ against ALL reflections. The weighted *R*-factor *wR* and goodness of fit *S* are based on *F*^2^, conventional *R*-factors *R* are based on *F*, with *F* set to zero for negative *F*^2^. The threshold expression of *F*^2^ > σ(*F*^2^) is used only for calculating *R*-factors(gt) *etc*. and is not relevant to the choice of reflections for refinement. *R*-factors based on *F*^2^ are statistically about twice as large as those based on *F*, and *R*- factors based on ALL data will be even larger.

### Fractional atomic coordinates and isotropic or equivalent isotropic displacement parameters (Å^2^)

**Table:**

	*x*	*y*	*z*	*U*_iso_*/*U*_eq_	
Se1	0.36683 (5)	0.57372 (4)	0.271792 (15)	0.02983 (13)	
Se2	0.41005 (5)	0.29253 (4)	0.487699 (15)	0.03145 (13)	
O1	0.3545 (5)	0.2664 (4)	0.43901 (12)	0.0799 (15)	
O2	0.5188 (4)	0.1974 (3)	0.50425 (14)	0.0553 (11)	
H2	0.5936	0.1963	0.4890	0.083*	
O3	0.5015 (5)	0.3935 (3)	0.49155 (15)	0.0644 (12)	
O4	0.2793 (4)	0.2887 (3)	0.52431 (10)	0.0452 (9)	
O5	0.2967 (4)	0.4638 (3)	0.27541 (12)	0.0459 (9)	
O6	0.4460 (4)	0.6107 (3)	0.31613 (11)	0.0476 (10)	
O7	0.4741 (4)	0.5824 (3)	0.22931 (11)	0.0554 (11)	
O8	0.2301 (4)	0.6563 (3)	0.26121 (17)	0.0647 (13)	
H8	0.1503	0.6299	0.2659	0.097*	
O9	1.1750 (4)	0.5569 (3)	0.38425 (14)	0.0651 (13)	
O10	1.1601 (4)	0.5021 (3)	0.45094 (14)	0.0589 (11)	
O11	−0.3916 (4)	0.2918 (3)	0.36698 (16)	0.0712 (14)	
O12	−0.3715 (4)	0.3419 (4)	0.29945 (16)	0.0713 (13)	
N1	0.7158 (4)	0.4848 (3)	0.44020 (13)	0.0345 (9)	
H1	0.6641	0.4587	0.4611	0.041*	
N2	0.5007 (4)	0.5204 (3)	0.40355 (15)	0.0420 (11)	
H2A	0.4529	0.4972	0.4259	0.050*	
H2B	0.4539	0.5430	0.3809	0.050*	
N3	1.1047 (5)	0.5295 (3)	0.41618 (15)	0.0419 (11)	
N4	0.0710 (4)	0.3519 (3)	0.31176 (13)	0.0358 (10)	
H4	0.1237	0.3769	0.2909	0.043*	
N5	0.2842 (4)	0.3237 (3)	0.35110 (14)	0.0436 (11)	
H5A	0.3340	0.3483	0.3294	0.052*	
H5B	0.3288	0.3025	0.3744	0.052*	
N6	−0.3196 (5)	0.3149 (3)	0.33474 (19)	0.0468 (12)	
C1	0.6453 (5)	0.5211 (3)	0.40398 (16)	0.0327 (11)	
C2	0.7316 (5)	0.5571 (3)	0.36909 (14)	0.0309 (11)	
H2C	0.6864	0.5780	0.3430	0.037*	
C3	0.8802 (5)	0.5620 (3)	0.37265 (15)	0.0328 (11)	
H3	0.9374	0.5874	0.3497	0.039*	
C4	0.9448 (5)	0.5275 (4)	0.41192 (16)	0.0311 (11)	
C5	0.8634 (5)	0.4879 (4)	0.44491 (16)	0.0342 (11)	
H5C	0.9080	0.4632	0.4704	0.041*	
C6	0.1403 (5)	0.3185 (3)	0.34854 (15)	0.0305 (10)	
C9	−0.1590 (5)	0.3146 (3)	0.33920 (17)	0.0330 (11)	
C8	−0.0953 (5)	0.2830 (3)	0.37952 (16)	0.0341 (11)	
H8C	−0.1541	0.2621	0.4030	0.041*	
C7	0.0523 (5)	0.2838 (3)	0.38333 (15)	0.0312 (11)	
H7	0.0960	0.2611	0.4093	0.037*	
C10	−0.0746 (5)	0.3482 (4)	0.30600 (16)	0.0356 (11)	
H10	−0.1167	0.3687	0.2794	0.043*	

### Atomic displacement parameters (Å^2^)

**Table:**

	*U*^11^	*U*^22^	*U*^33^	*U*^12^	*U*^13^	*U*^23^
Se1	0.0236 (2)	0.0403 (3)	0.0256 (2)	−0.0052 (2)	0.00113 (19)	−0.0015 (2)
Se2	0.0267 (2)	0.0409 (3)	0.0268 (2)	−0.0001 (2)	0.00137 (19)	0.0081 (2)
O1	0.061 (3)	0.148 (5)	0.030 (2)	0.001 (3)	−0.006 (2)	−0.004 (3)
O2	0.035 (2)	0.051 (2)	0.080 (3)	0.0063 (18)	0.0125 (19)	0.029 (2)
O3	0.070 (3)	0.039 (2)	0.084 (3)	−0.015 (2)	0.034 (2)	0.000 (2)
O4	0.0268 (19)	0.076 (3)	0.0327 (19)	−0.0044 (18)	0.0055 (14)	0.0082 (19)
O5	0.045 (2)	0.038 (2)	0.054 (2)	−0.0077 (17)	0.0136 (18)	−0.0064 (18)
O6	0.052 (2)	0.061 (3)	0.0296 (19)	−0.007 (2)	−0.0080 (17)	−0.0101 (18)
O7	0.035 (2)	0.100 (3)	0.0312 (19)	−0.023 (2)	0.0111 (16)	−0.006 (2)
O8	0.029 (2)	0.049 (2)	0.117 (4)	−0.0035 (18)	−0.009 (2)	0.025 (2)
O9	0.033 (2)	0.105 (4)	0.058 (3)	−0.007 (2)	0.012 (2)	0.002 (3)
O10	0.039 (2)	0.075 (3)	0.062 (3)	0.008 (2)	−0.015 (2)	0.004 (2)
O11	0.033 (2)	0.086 (4)	0.094 (4)	−0.004 (2)	0.025 (2)	0.012 (3)
O12	0.031 (2)	0.093 (4)	0.091 (4)	0.002 (2)	−0.016 (2)	0.016 (3)
N1	0.031 (2)	0.041 (2)	0.032 (2)	−0.0018 (19)	0.0058 (17)	0.0056 (19)
N2	0.025 (2)	0.057 (3)	0.044 (3)	−0.003 (2)	0.0021 (18)	0.007 (2)
N3	0.033 (3)	0.046 (3)	0.047 (3)	0.004 (2)	0.001 (2)	−0.007 (2)
N4	0.028 (2)	0.050 (3)	0.029 (2)	−0.0023 (19)	−0.0010 (17)	0.0121 (19)
N5	0.031 (2)	0.059 (3)	0.040 (2)	−0.005 (2)	−0.0055 (18)	0.008 (2)
N6	0.030 (2)	0.032 (3)	0.079 (4)	0.0035 (19)	−0.003 (3)	0.000 (2)
C1	0.038 (3)	0.024 (2)	0.036 (3)	0.001 (2)	0.000 (2)	−0.004 (2)
C2	0.030 (3)	0.039 (3)	0.024 (2)	0.002 (2)	0.000 (2)	0.002 (2)
C3	0.034 (3)	0.031 (3)	0.033 (3)	0.001 (2)	0.007 (2)	0.001 (2)
C4	0.027 (3)	0.034 (3)	0.033 (3)	0.001 (2)	0.000 (2)	−0.006 (2)
C5	0.035 (3)	0.035 (3)	0.032 (3)	0.002 (2)	−0.008 (2)	−0.001 (2)
C6	0.028 (2)	0.030 (3)	0.034 (3)	−0.001 (2)	−0.006 (2)	−0.003 (2)
C9	0.027 (3)	0.027 (3)	0.046 (3)	−0.001 (2)	0.000 (2)	−0.004 (2)
C8	0.039 (3)	0.030 (3)	0.033 (3)	0.000 (2)	0.012 (2)	−0.004 (2)
C7	0.037 (3)	0.032 (3)	0.024 (2)	0.002 (2)	−0.004 (2)	0.001 (2)
C10	0.033 (3)	0.041 (3)	0.034 (3)	−0.002 (2)	−0.010 (2)	0.010 (2)

### Geometric parameters (Å, °)

**Table:**

Se1—O6	1.597 (3)	N4—C10	1.337 (6)
Se1—O5	1.610 (3)	N4—C6	1.352 (6)
Se1—O7	1.614 (3)	N4—H4	0.8600
Se1—O8	1.696 (4)	N5—C6	1.312 (6)
Se2—O1	1.592 (4)	N5—H5A	0.8600
Se2—O3	1.594 (4)	N5—H5B	0.8600
Se2—O4	1.623 (3)	N6—C9	1.466 (6)
Se2—O2	1.690 (4)	C1—C2	1.398 (6)
O2—H2	0.8200	C2—C3	1.357 (6)
O8—H8	0.8200	C2—H2C	0.9300
O9—N3	1.213 (5)	C3—C4	1.400 (6)
O10—N3	1.220 (5)	C3—H3	0.9300
O11—N6	1.213 (6)	C4—C5	1.349 (7)
O12—N6	1.219 (6)	C5—H5C	0.9300
N1—C5	1.350 (6)	C6—C7	1.399 (6)
N1—C1	1.356 (6)	C9—C10	1.339 (7)
N1—H1	0.8600	C9—C8	1.412 (7)
N2—C1	1.315 (6)	C8—C7	1.347 (6)
N2—H2A	0.8600	C8—H8C	0.9300
N2—H2B	0.8600	C7—H7	0.9300
N3—C4	1.459 (6)	C10—H10	0.9300
			
O6—Se1—O5	113.95 (19)	O12—N6—C9	117.8 (5)
O6—Se1—O7	111.69 (19)	N2—C1—N1	118.5 (5)
O5—Se1—O7	111.02 (19)	N2—C1—C2	123.8 (5)
O6—Se1—O8	106.5 (2)	N1—C1—C2	117.7 (5)
O5—Se1—O8	108.74 (19)	C3—C2—C1	121.1 (5)
O7—Se1—O8	104.3 (2)	C3—C2—H2C	119.5
O1—Se2—O3	114.9 (3)	C1—C2—H2C	119.5
O1—Se2—O4	112.8 (2)	C2—C3—C4	117.9 (4)
O3—Se2—O4	111.1 (2)	C2—C3—H3	121.0
O1—Se2—O2	107.0 (3)	C4—C3—H3	121.0
O3—Se2—O2	108.3 (2)	C5—C4—C3	121.5 (4)
O4—Se2—O2	101.76 (18)	C5—C4—N3	119.3 (4)
Se2—O2—H2	109.5	C3—C4—N3	119.1 (4)
Se1—O8—H8	109.5	C4—C5—N1	118.7 (4)
C5—N1—C1	122.9 (4)	C4—C5—H5C	120.6
C5—N1—H1	118.6	N1—C5—H5C	120.6
C1—N1—H1	118.6	N5—C6—N4	119.7 (4)
C1—N2—H2A	120.0	N5—C6—C7	123.0 (4)
C1—N2—H2B	120.0	N4—C6—C7	117.3 (4)
H2A—N2—H2B	120.0	C10—C9—C8	120.7 (4)
O9—N3—O10	123.7 (5)	C10—C9—N6	120.0 (5)
O9—N3—C4	117.4 (4)	C8—C9—N6	119.2 (5)
O10—N3—C4	118.8 (4)	C7—C8—C9	118.7 (4)
C10—N4—C6	123.8 (4)	C7—C8—H8C	120.7
C10—N4—H4	118.1	C9—C8—H8C	120.7
C6—N4—H4	118.1	C8—C7—C6	120.6 (4)
C6—N5—H5A	120.0	C8—C7—H7	119.7
C6—N5—H5B	120.0	C6—C7—H7	119.7
H5A—N5—H5B	120.0	N4—C10—C9	118.9 (4)
O11—N6—O12	124.5 (5)	N4—C10—H10	120.6
O11—N6—C9	117.6 (5)	C9—C10—H10	120.6
			
C5—N1—C1—N2	−175.8 (4)	C10—N4—C6—N5	179.0 (5)
C5—N1—C1—C2	4.3 (7)	C10—N4—C6—C7	−4.0 (7)
N2—C1—C2—C3	175.6 (5)	O11—N6—C9—C10	−173.8 (5)
N1—C1—C2—C3	−4.5 (7)	O12—N6—C9—C10	3.5 (7)
C1—C2—C3—C4	1.6 (7)	O11—N6—C9—C8	3.1 (7)
C2—C3—C4—C5	1.8 (7)	O12—N6—C9—C8	−179.5 (5)
C2—C3—C4—N3	178.4 (4)	C10—C9—C8—C7	−2.5 (7)
O9—N3—C4—C5	173.2 (5)	N6—C9—C8—C7	−179.5 (4)
O10—N3—C4—C5	−5.4 (7)	C9—C8—C7—C6	2.2 (7)
O9—N3—C4—C3	−3.4 (7)	N5—C6—C7—C8	177.8 (5)
O10—N3—C4—C3	177.9 (5)	N4—C6—C7—C8	0.9 (7)
C3—C4—C5—N1	−2.2 (7)	C6—N4—C10—C9	3.7 (8)
N3—C4—C5—N1	−178.8 (4)	C8—C9—C10—N4	−0.3 (8)
C1—N1—C5—C4	−1.0 (7)	N6—C9—C10—N4	176.6 (5)

### Hydrogen-bond geometry (Å, °)

**Table:**

*D*—H···*A*	*D*—H	H···*A*	*D*···*A*	*D*—H···*A*
O2—H2···O4^i^	0.82	1.75	2.527 (5)	158
O8—H8···O7^ii^	0.82	1.73	2.546 (5)	173
N1—H1···O3	0.86	1.95	2.773 (5)	161
N2—H2A···O3	0.86	2.46	3.152 (6)	138
N2—H2B···O6	0.86	2.15	2.943 (6)	152
N2—H2B···O9^iii^	0.86	2.54	3.057 (6)	119
N4—H4···O5	0.86	2.01	2.769 (5)	146
N5—H5A···O5	0.86	2.27	2.958 (6)	137
N5—H5B···O1	0.86	2.02	2.833 (6)	157
N5—H5B···O11^iv^	0.86	2.56	3.016 (6)	115
C2—H2C···O6	0.93	2.37	3.132 (6)	139
C8—H8C···O4^v^	0.93	2.37	3.261 (6)	159
C3—H3···O7^vi^	0.93	2.41	3.202 (6)	143
C5—H5C···O2^i^	0.93	2.50	3.245 (6)	137
C5—H5C···O10^vii^	0.93	2.50	3.150 (6)	128
C7—H7···O1	0.93	2.52	3.228 (6)	134
C10—H10···O5^ii^	0.93	2.23	3.130 (6)	162

Symmetry codes: (i) *x*+1/2, −*y*+1/2, −*z*+1; (ii) *x*−1/2, *y*, −*z*+1/2; (iii) *x*−1, *y*, *z*; (iv) *x*+1, *y*, *z*; (v) *x*−1/2, −*y*+1/2, −*z*+1; (vi) *x*+1/2, *y*, −*z*+1/2; (vii) −*x*+2, −*y*+1, −*z*+1.
